# Readiness for Perception and Action: Towards a More Mechanistic Understanding of Phasic Alertness

**DOI:** 10.5334/joc.426

**Published:** 2025-01-21

**Authors:** Christian H. Poth

**Affiliations:** 1 Neuro-Cognitive Psychology, Department of Psychology, Bielefeld University, Bielefeld, Germany

**Keywords:** Arousal, attention, temporal expectation, vision, audition, decision

## Abstract

Human survival requires prompt perception and action to address relevant events in the environment. For this, the brain has evolved a system that uses warning stimuli to elicit phasic alertness, a state readying the brain for upcoming perception and action. Although a wealth of empirical evidence revealed how phasic alertness improves a wide range of perceptual and cognitive processing, it is still unclear by what cognitive mechanisms this is achieved. Here, we identify key problems that have to be solved for this to be possible and delineate concrete ways to achieve this. Specifically, we discover I) how to establish phasic alertness as a cognitive state of readiness for perception and action, II) how it can affect cognition online or offline, III) how it could be triggered internally without a warning, and IV) to what degrees it relied on bottom-up processing, or top-down temporal or stimulus expectations and the current task. As a result, the discussion provides us with a research program yielding the theoretical and empirical basis for mechanistic and computational models of phasic alertness and its neurophysiological underpinnings.

In many situations, humans must react promptly to events in the environment. Eliciting action on time poses a challenge for the brain mechanisms underlying perception and action. To prevent that responses to events come too late, the events have to be accommodated in perceptual and cognitive processing as early as possible. To address this challenge, the brain has evolved a *pre-warning* or *alerting system* assumed to support fast perception and action by readying their underlying mechanisms already before the events take place based on stimuli preceding them (S. E. Petersen & Posner, 2012; Posner & Petersen, 1990; Sturm & Willmes, 2001). Even though the beneficial effects of alerting on behavior have been documented extensively (see below), it is still unclear by which cognitive mechanisms alerting is achieved. Here, we first provide a selective overview of a number of findings from the literature on phasic alertness, specifically focusing on our own research lines from the last years. On this basis, we develop a specific perspective on the research field of phasic alertness that leads us to discuss a number of key challenges to be overcome for understanding phasic alertness more mechanistically in the future. We also develop concrete research strategies by which this can be achieved. However, to do right by the rich literature on phasic alertness, it should be noted that there are a number of aspects of phasic alertness that are beyond the scope of our limited and selective discussion (such as the rich literature on phasic alertness and its interplay with cognitive control and cognitive conflicts, Callejas et al., 2005; Correa et al., 2010; Fan et al., 2002; Fischer et al., 2010; MacLeod et al., 2010; McConnell & Shore, 2011; Meiran & Chorev, 2005; D. W. Schneider, 2018; 2019a; 2019b; 2020; Weinbach & Henik, 2012b; Kahan & Zhang, 2019; Seibold, 2018).

## Phasic vs. tonic alertness: Two types of readiness for perception and action

Alertness describes a human’s current state of readiness for perception and action (S. E. Petersen & Posner, 2012; Posner, 1978; 2008; Posner & Petersen, 1990). Two forms of alertness are commonly distinguished (Sturm & Willmes, 2001). Tonic alertness refers to the degree of readiness for perception and action that varies over intermediate time-scales, ranging from minutes to hours (Oken et al., 2006; Sturm & Willmes, 2001). Tonic alertness is assumed to be intrinsic in the sense that its variation is not due to activation by external stimuli. Its variation can be seen in monitoring tasks (Poth et al., 2014), in which performance declines over time (the vigilance decrement, e.g., Hancock, 2017; See et al., 1995) or fluctuates from time to time (Esterman et al., 2014; Fortenbaugh et al., 2017). In contrast to tonic alertness, phasic alertness refers to the increase of readiness for perception and action for brief periods (in the range of hundreds of milliseconds to a few seconds) that is induced by an external stimulus (an alerting cue or warning stimulus; Fan et al., 2002; 2005; Hackley, 2009; Posner & Boies, 1971).

### Phasic alertness and behavior

Phasic alertness becomes manifest in the so-called alerting effect: Reaction times in choice-tasks are improved if target stimuli are preceded within about 500 ms by alerting cues (Bernstein et al., 1969; Fan et al., 2002; 2005; Hackley, 2009; Jepma et al., 2009; Keuss et al., 1990; Poth, 2020). This benefit happens, even though the alerting cues offer no information about what response has to be made. Thus, rather than providing specific information about the response, alerting seems to affect processing for perception and action more broadly (for a limited time). That is, alerting is assumed to change the current alertness state of the cognitive system, so that different parts of processing become faster and/or are improved otherwise (S. E. Petersen & Posner, 2012; Posner, 1978; Posner & Boies, 1971; Posner & Petersen, 1990). This idea of phasic alertness as a short-lived state-change for the cognitive system rather than a modulation of a specific process is underscored by the diversity of processes that are affected by alerting. Alerting has been found to increase perceptual sensitivity in tasks with no speed requirements (Kusnir et al., 2011), to increase the temporal resolution of perception (Li et al., 2018), to make visual processing earlier and faster for encoding objects into short-term memory for perception and action control (Haupt et al., 2019; Matthias et al., 2010; A. Petersen et al., 2017; Wiegand et al., 2017), to speed up visual search (Dietze & Poth, 2024; Jankovic et al., 2022) and the first action in sensorimotor sequences (Dietze et al., 2023).

The benefits of alerting for fast perception and action sometimes come at the cost of more frequent behavioral errors (speed-accuracy-tradeoff; Dietze & Poth, 2022; Han & Proctor, 2022; McCormick et al., 2019; Posner et al., 1973). Also, alerting can sometimes have disadvantages for goal-driven behavior when this conflicts with action tendencies elicited by stimuli in the environment. That is, alerting might increase cognitive conflicts (or respectively, interference) between stimulus-driven and goal-driven behavior (by several potential mechanisms, such as spatial and non-spatial mechanisms), so that the typical response slowing due to conflicting stimuli (so-called congruency effects) can be modulated or increased (Callejas et al., 2005; Correa et al., 2010; Fan et al., 2002; Fischer et al., 2010; Kahan & Zhang, 2019; MacLeod et al., 2010; McConnell & Shore, 2011; Meiran & Chorev, 2005; D. W. Schneider, 2018; 2019a; 2019b; 2020; Seibold, 2018; Weinbach & Henik, 2012b).

Taken together, the advantageous and disadvantageous effects of alerting on perception and action suggest that phasic alertness puts the cognitive system in a temporary state in which fast perception and action are supported rigorously. This happens even at the expense of errors and conflicts between goal-driven and stimulus-driven behavior. The multitude of tasks and their required cognitive functions that are affected by alerting suggest that alerting is unspecific, process- and task-general. This would be in line with phasic alerntness as a broad state change of the cognitive system. Critically, however, whether or not alerting really leads to such a state change remains a fundamental yet open question (see below).

## Challenges of understanding phasic alertness effects on cognition

### Phasic alertness as attentional intensity and its distinction from selective attention

As a temporary state of the cognitive system, phasic alertness (and alertness in general) refers to the intensity aspect of attention that we have to distinguish from selective attention (see Bundesen et al., 2015, for a related discussion). Attention refers to the mechanisms that set priorities in information processing and allocate specific processing resources accordingly (Bundesen, 1990; Bundesen et al., 2005; Desimone & Duncan, 1995; Duncan & Humphreys, 1989; Treisman & Gelade, 1980; Wolfe, 1994; 2021). It is a longstanding question, whether the cognitive mechanisms underlying phasic alertness can be dissociated from those that support selective and divided attention.

Phasic alertness and selective spatial attention have long been assumed to be implemented by different “attentional networks” of the brain that were separate but could interact (Fan et al., 2002; 2005; S. E. Petersen & Posner, 2012; Posner & Petersen, 1990). Indeed, behavioral alerting effects have sometimes been found to interact with effects of spatial attention (Asanowicz & Marzecová, 2017; Callejas et al., 2005; Festa-Martino et al., 2004; Fuentes & Campoy, 2008; Karpouzian-Rogers et al., 2020; Lin & Lu, 2016), or induce spatial biases (Chandrakumar et al., 2019), suggesting that phasic alertness may speed up the allocation of attention in space. However, sometimes no interactive relationship has been found and instead, alerting and spatial orienting had additive effects on behavior (Botta et al., 2014; Chica et al., 2011). Moreover, no alerting effects have been observed that modulated selective attention for object recognition (Ásgeirsson & Nieuwenhuis, 2017). Here, attention was based on the surface features (i.e. color) of objects rather than the object’s spatial location (Ásgeirsson & Nieuwenhuis, 2017). At the level of underlying cognitive mechanisms, alerting seems unaffected by the task sets delivering the priorities for controlling spatial attention. This suggests that the sources of top-down control differ at least partially for phasic alertness and spatial attention (Dietze & Poth, 2022).

It has been hypothesized that cognitive states such as alertness could interface with attention mechanisms by modulating attentional biases that scale the processing of visual features (Bundesen et al., 2015). In this view, selective attention should work most effectively at intermediate levels of alertness, approaching ineffective attentional selection for extremely low and extremely high levels of alertness (akin to floor and ceiling effects of attentional selection at these extreme levels of alertness, cf. Bundesen et al., 2015). Thus, the effectiveness of selective attention should be an inverse U-shaped function of the current alertness level (akin to the Yerkes-Dodson law, Yerkes & Dodson, 1908). This idea dovetails with classic suggestions that selective attention presupposes a minimum level of alertness (Posner, 1978), but remains to be tested experimentally.

#### Where is the locus of alerting effects in the cognitive processing chain?

Decades of research have been devoted to the quest of finding the locus of alerting (or warning signal effects) within the chain of cognitive processing. Some accounts posited that alerting reduced reaction times in simple decision tasks by speeding up motor processing and response execution (Posner, 1978; Sanders, 1980). Other accounts suggested perceptual processing (Jepma et al., 2009; Rolke, 2008) or later perceptual and response selection processes as cognitive loci of the alerting effect on reaction time (Fischer et al., 2010; Hackley, 2009; Hackley & Valle-Inclán, 1998; 1999; 2003). It was long thought that this issue had been settled with studies that partitioned reaction times according to components in the electroencephalogram (the lateralized readiness potential). These studies showed that processes before motor processes of response execution were shortened by alerting (Hackley & Valle-Inclán, 1998). Based on these findings, it had been assumed that phasic alertness elicits its effects by speeding up (“late”) perceptual processes and/or response selection (Hackley, 2009). More recent work demonstrated that alerting also affected early perceptual encoding of visual information. That is, alerting increased the sensitivity of visual perception (Kusnir et al., 2011), speeded up visual processing for encoding into visual short-term memory, and led to an earlier start of visual processing (Haupt et al., 2019; 2021; Matthias et al., 2010; A. Petersen et al., 2017; Wiegand et al., 2017). As such, these findings provide unequivocal evidence that alerting also affects perceptual processing that is assumed to happen before response selection. We should note, however, that this does not mean that alerting solely affects perceptual encoding. As explained above, alerting affects a wide range of cognitive functions, and these functions could be provided by mechanisms at different processing phases or stages. Thus, phasic alertness seems to propagate through the entire cognitive system (e.g., by means of modulation of cortical processing by norepinephrine (NE), Posner & Petersen, 1990, see below). The sizes of alerting effects at different phases or stages of processing may differ, and could combine so that the largest effect emerged at the response selection stage (not later, as alerting effects seem to happen before the motor state, see above Hackley & Valle-Inclán, 1998).

The described “processing-stage accounts” attribute alerting effects to an influence on one or (simultaneously) more of several stages of processing (for instance, stages such as early visual processing, figure-ground segregation, attention-dependent encoding into visual working memory, response selection; see, Bundesen et al., 2005; Pashler, 1994; Treisman & Gelade, 1980, for some examples of stage-based processing models). For example, alerting could selectively shorten the time required for processing at the response selection stage (see above). In contrast to processing-stage accounts, one may also suppose that alerting influenced all cognitive processing for a certain time-window after the alert, irrespective of what process was currently active and irrespective of the processing stage at which the process was located (Jankovic et al., 2022). This idea seems in line with the proposal that the phasic noradrenaline release that could underlie phasic alertness implemented a temporal filter that amplified processing in a time-window (Aston-Jones & Cohen, 2005). The idea also fits to the conception of phasic alertness as non-specific with respect to specific cognitive processes, which matches the diversity of cognitive functions that have been found modulated by alerting (see above, section *Phasic alertness and behavior*). That is because the cognitive functions should differ in the processing stages of their underlying mechanisms, so that it would seem unlikely that a single stage accounted for the diversity of effects. What is problematic, however, is that the time-based account seems difficult to test empirically. For instance, a recent study investigated how alerting affected performance in a simple visual search task supposed to mainly require target detection as compared with a more complex compound visual search task supposed to require target detection and discrimination (Jankovic et al., 2022). This study found alerting effects only for the simple and not for the complex task. This was interpreted in favor of the time-based account. For simple tasks, phasic alertness sped up processing for detection. In contrast, for the complex task, detection was sped up by phasic alertness as well. However, the modulation by phasic alertness was short-lasting, and so that a supposed additional and subsequent process of task-set reconfiguration that was necessary for the discrimination was not sped up. Now, it was assumed that this process was sped up when there was no alert, because the onset of the visual search stimuli themselves induced phasic alertness. Therefore, the speeding up of the detection and the speeding up of task-set reconfiguration for discrimination canceled out, resulting in the absence of alerting effects. What argues against these hypotheses, are opposing findings showing that alerting in fact also improved visual search performance in highly difficult compound visual search tasks (Dietze & Poth, 2024). In addition, we should note that the described processes of detection and discrimination as sequential operations are not the only way to conceptualize attention in visual search, and may also be realized within the same process in parallel (Bundesen, 1990).

Based on the above considerations, we can arrive at the opinion that both studies remain insufficient for testing the time-based account of phasic alertness. This also points us at a general problem of assuming sequential processing stages on the one hand and time-based effects on the other hand. That is, when we assume processing stages, for example, target detection, task-set reconfiguration, and target discrimination, in visual search, we typically leave their durations unspecified. Thus, as in the example of the compound visual search task, alerting by the alerting cue may speed up one process, whereas the onset of the search stimuli may speed up another process, so that both improvements cancel out. Therefore, it seems necessary to independently manipulate or measure the durations of the different processing stages, in order to be able to quantify whether or not such a cancelling-out had taken place. Only this would allow to interpret null effects of alerting as evidence for time-based accounts. In other words, finding alerting effects in different tasks that rely on different processing stages cannot alone decide between processing-stage or time-based accounts: Even if the time-based account was true, alerting effects (rather than null effects) should still emerge even in more complex tasks that involve more processing stages. If one assumes that the onset of a stimulus such as the search display constituted a kind of sensory transient in the input stream changing the level of alertness, and if one assumes that the timing of this effect differed from that of the alerting effect induced by the alerting cue, then the two effects may not cancel out each other in the comparison of no-cue and alerting-cue condition. One way to approach this problem may be to vary alerting-cue-target onset asynchronies (CTOAs) and alerting cue intensities randomly, and then reverse-correlate their effects with task performance (cf. Okazawa et al., 2018). However, this endeavor may be non-trivial, because, as argued below (see section *Phasic alertness and top-down temporal preparation: Distinct processes?*), the probability distributions chosen for this random variation may greatly affect the results by inducing their own effects on perceptual and cognitive processing (e.g., by inducing temporal expectations, Vangkilde et al., 2012; 2013; Weinbach & Henik, 2012a) or threshold adjustment to the mean intensities across trials (Levison & Restle, 1968).

To conclude, given the diversity of cognitive functions that are affected by alerting and the hierarchical organization of perceptual and cognitive processing, it seems unlikely that there was a single locus in the processing chain at which alerting effects arise. Rather, it seems more likely that alerting effects arise at multiple levels of processing and propagate forward through the levels of processing.

#### Does phasic alertness affect perception and cognition online or offline?

**Figure 1:**
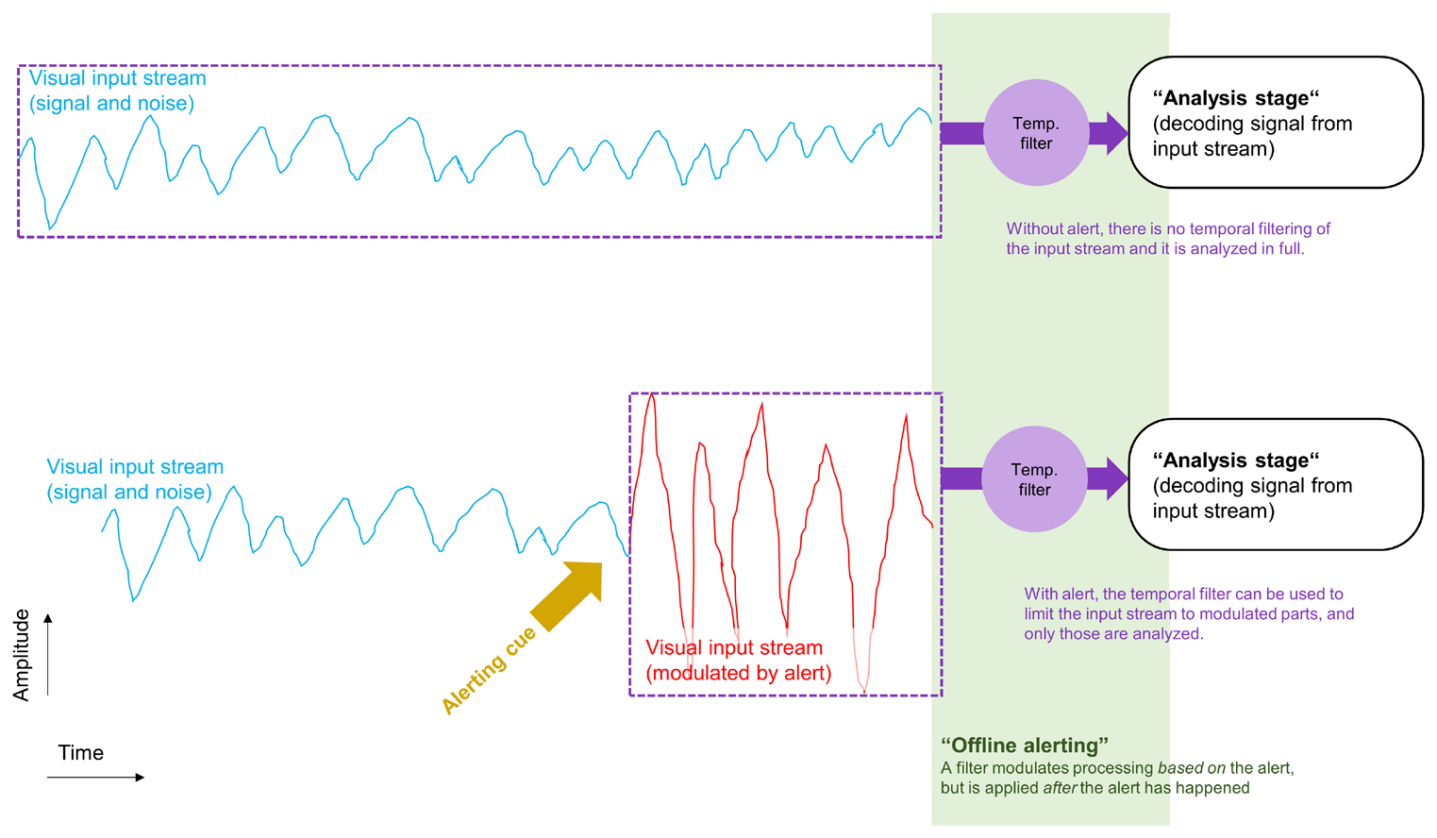
Illustration of hypothetical influences of alerting that happen “offline”, at a later processing stage. A visual input stream consisting in a signal and noise over time is encoded and processed by a later “analysis stage” whose processes aim to decode the signal from the input stream (upper panel). Usually, the complete input stream is analyzed. However, if there is an alerting cue, the input stream (the signal and noise) are modulated (lower panel). Now, a temporal selection process before the analysis stage filters-out the non-modulated parts of the input stream, so that only a small part of the input stream that deviates from the rest (e.g. in relative amplitude) has to be analyzed later on at the analysis stage.

The current notion of phasic alertness is that it is a temporary state of readiness for perception and action (S. E. Petersen & Posner, 2012; Posner & Petersen, 1990; Sturm & Willmes, 2001). This means that being phasically alert *prepared* the cognitive system for perception and action. The state of phasic alertness was reached first, and exerted its effects prospectively by modulating the cognitive processing that followed (Brown et al., 2015; Hackley & Valle-Inclán, 2003; Jepma et al., 2009; Tona et al., 2016). Therefore, the current state of alertness should influence ongoing cognition in an online fashion. Phasic alertness as an online-influence on cognition seems intuitive because the sequence of cognitive processing would match the sequence of events in experimental paradigms: I) The alert induced phasic alertness, II) phasic alertness modulated current cognition (online), III) impacting on the processing of target stimuli and responding within the task. Critically, however, we should also consider that the timing and sequence of events in the experimental paradigm need not be the same as the timing and sequence of cognitive events happening in the brain. Thus, in addition to prospective influences on cognition that happened in an online-fashion, phasic alerting may also have offline effects on perception and cognition. Specifically, alerts could in some way be encoded with the information about the target stimuli of the task and then affect their processing later on, offline, once they are decoded for selecting a response, for example. For instance, alerts (simultaneous accessory stimuli) can reduce the temporal uncertainty of target stimuli that are to be reported later on (Lippert et al., 2007). We could imagine that the target stimuli of a task are encoded as a signal in a temporal stream of information and noise (see Figure 1, upper panel). Now, an alert could be encoded in some way within this stream as well. For example, the alert could modulate the input stream by increasing its signal or noise amplitude (see Figure 1, lower panel) or by adding noise. The perceptual analysis of the input stream happens later on, after initial encoding, and after the modulation of the input stream by the alert. Now, before the input stream is analyzed perceptually to extract the signal from the stream, it is processed by a temporal filter (cf. Aston-Jones & Cohen, 2005, as discussed below). The filter could be set in a way that lets through only those parts of the input stream that deviated from the rest of the input stream according to their temporal and intensity characteristics. So, when the alert increased the amplitude of the input stream (Figure 1, lower panel, red graph), it enabled the later filter to restrict the later perceptual analysis to only a part of the input stream, rather than requiring analysis of the full stream. Such an influence of alerting on cognitive processing would be “offline”, in the sense that the filter was applied to the input stream after the alert, and then affected the efficiency of the subsequent perceptual analysis stage, but did not affect the computational processes at this stage directly (only passively, by modulating the input stream beforehand, Figure 1). Thus, the alert would be similar to a temporal landmark allowing to temporally filter the signal (cf. Aston-Jones & Cohen, 2005), exclude parts of the stream that are unnecessary, and focus computationally demanding perceptual processing only on the interval after the alert. This would explain findings that two successive alerts (i.e. an alert and a so-called accessory stimulus appearing simultaneously with the target) offered smaller benefits for choice reaction than a single alert (Poth, 2020). The two alerts could force the analysis stage to consider two parts of the input stream, increasing the amount of input that needs to be analyzed and induce some temporal uncertainty regarding where in the input stream the target signal could be found. This would then decrease the benefits from the alerting cues as compared to a situation with only one alerting cue. In addition, if the two parts of the input stream were represented separately at the perceptual analysis stage, there could also arise a competition between them for processing, calling for attention mechanisms allocating available processing resources to the parts (Bundesen, 1990; Bundesen et al., 2005). Prioritizing each part equally would lead to a situation of divided attention (Bundesen, 1990; Bundesen et al., 2005), compared with a situation of only one alert, this would mean that the additional alert would be “distracting”, in the sense that it consumed processing capacity that would otherwise be available for processing the other part. Thus, even though it seems reasonable to assume that alerting worked prospectively in an online-fashion (S. E. Petersen & Posner, 2012; Posner & Petersen, 1990; Sturm & Willmes, 2001), it seems fruitful to speculate about potential offline-effects on subsequent perception and cognition, as this forces us to consider the role of the processing architecture (i.e. which temporal filter is applied at what stage of processing). This idea is in line with recent calls for entertaining both, prospective and retrospective thoughts about cognition more generally, rather than restricting models to one of these alternatives (Herzog et al., 2020; Hogendoorn, 2022).

### What mechanisms elicit phasic alertness?

Now that we have established what phasic alertness is, we can move on to discuss its mechanistic basis, specifically how it could be elicited based on the current state of the arousal system and what types of candidate mechanisms may underlie and support it. This discussion reflects the current state of model building that already provides verbal and neurophysiologically-based descriptions of candidate mechanisms (e.g., Aston-Jones & Cohen, 2005; Posner & Petersen, 1990; 2012), but has not yet arrived at mechanistic models that mathematically specify the computations happening over time (so-called computational process models, as are available for visual attention, e.g., Bundesen, 1990).

#### Extrinsic stimulus-driven versus intrinsic phasic alertness

**Figure 2:**
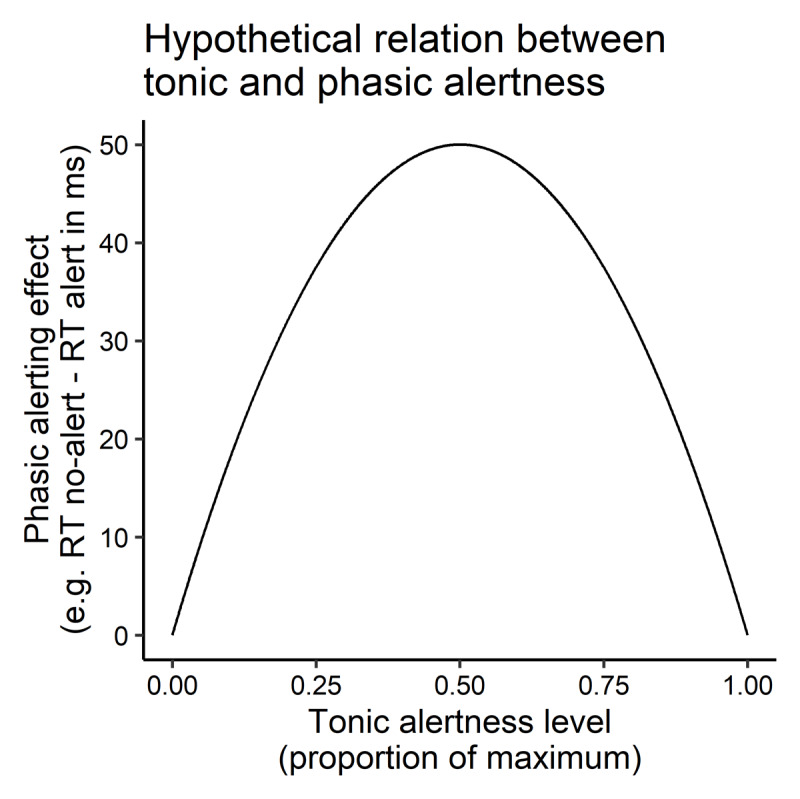
Hypothetical (inverse U-shaped) relationship between the current level of tonic alertness and the effects of phasic alertness on perceptual and cognitive processing and behavioral performance (e.g., alerting effects on reaction times in ms in choice tasks).

The effects of phasic alerting on other cognitive processes should depend on the baseline level of tonic alertness, and this relationship should be nonlinear, for instance, inverse U-shaped. Alerting effects should be large if tonic alertness had been intermediate, but should be small if tonic alertness had been low or high (Figure 2).

This is in line with commonly held views of the neurophysiological underpinnings of phasic alertness. Alertness is assumed to rely on cortical arousal that is modulated by the the locus-coeruleus (LC) norepinephrine system (Aston-Jones & Cohen, 2005; S. E. Petersen & Posner, 2012; Posner & Petersen, 1990). The LC has widespread connections to cortical neurons implementing diverse cognitive functions, which makes the LC-NE system a candidate for underlying the various behavioral effects of alerting (McBurney-Lin et al., 2019; Poe et al., 2020; Sara, 2009). Alerting is thought to result in a brief release of norepinephrine by the LC in the brain stem (e.g., Brown et al., 2015; Coull et al., 2001; Tona et al., 2016). The LC-NE system seems to operate in two different modes, a tonic and a phasic mode (Aston-Jones & Cohen, 2005). The tonic mode of LC-NE activity sets the baseline norepinephrine release (Aston-Jones & Cohen, 2005), and at the level of the cognitive system, this baseline may correspond to tonic alertness. Tonic LC-NE activity has been found to relate to cognitive performance in a nonlinear, inverted-U-shaped function, with poor performance for low and high tonic activity, and highest performance at intermediate activity levels (Aston-Jones et al., 1999). In this way, the relationship follows the classic Yerkes-Dodson-Law (Aston-Jones & Cohen, 2005; Yerkes & Dodson, 1908), as an instance of the more general principle of hormesis biology and pharmacology (Calabrese, 2008). In the phasic mode, short bursts of NE release happen time-locked to the decision processes involved in the current task, which supports task performance (Aston-Jones & Cohen, 2005). Specifically, the bursts of NE release could implement a temporal attention filter that is general and non-specific to given processes and works by enhancing just the current processing for the task (Aston-Jones & Cohen, 2005). The pattern of phasic and tonic LC-NE activity resembles the relationship between phasic and tonic alertness described above: At low and at high tonic activity, phasic bursts of NE release cannot be observed, thus preventing any phasic modulation of NE at floor or ceiling levels. In contrast, at intermediate levels of tonic activity, phasic burst of NE become visible and could affect perception and action. Thus, taken together, and according to this assumption of a common mechanistic basis of phasic and tonic alertness, the effects of phasic alertness at the behavioral and the neurophysiological level should directly depend on the current level of tonic alertness (see above). Currently, however, this problem is mostly ignored by research on alerting. Studies on phasic alerting use experimental paradigms that neither assess nor explicitly manipulate participants’ current level of tonic alertness and instead only measure the effect of the alerting manipulation (cf. Figure 2, see the studies in section *Phasic alertness and behavior*) (but see Asanowicz & Marzecová, 2017; Martella et al., 2014; even though experimental paradigms exist that combine phasic alerting and measures of tonic alertness in the sense of vigilance, Luna et al., 2018). Thus, most studies are unable to detect where participants are on the inverted-U function that relates alerting effects to the level of tonic alertness (Figure 2). It is important to consider when this would be a problem for studying phasic alertness and when it would be of little concern. It would be problematic, when the source of low or high tonic alertness was located in the experiment. At high levels of tonic alertness, the effects of alerting compared with situation without alerting could be concealed. This could be the case in experiments involving challenging tasks or in which a challenging task is required in addition. Likewise, at low levels of tonic alertness, such as in situations with a low overall stimulation and low frequency of events (e.g., Hancock, 2017) alerting effects may likewise be concealed, either because processing happens at such a low level that it cannot be scaled by alerting (cf. Bundesen et al., 2015 for a similar idea for attention) or because the alert would come as a surprisingly strong stimulus that may inhibit responding and thereby hinder the manifestation of alerting effects in behavior (Dietze et al., 2024, see below). The potential effects of tonic alertness on phasic alerting effects would be of smaller concern, if the source of tonic alertness resided in individual participants (e.g. persons with naturally higher or lower tonic alertness as a personal trait), as long as phasic alerting effects are assessed within subjects or groups for between-subjects designs are large enough and randomly sampled from the population. As mentioned above, phasic alertness not only means that alertness is increased *phasically*, that is, in a short-lived fashion. Rather, a sometimes neglected feature of of phasic alertness is that it is defined to occur in response to an eliciting external stimulus (Sturm & Willmes, 2001). For this reason, phasic alertness is also called *extrinsic* alertness, as opposed to *intrinsic* alertness that is used synonymously with tonic alertness (Sturm & Willmes, 2001). These distinctions are problematic for several reasons. First, the tonic intrinsic alertness and phasic extrinsic alertness confound the time-scale of variation (longer vs. shorter) with the source of alertness (internal vs. external). Thus, by using this definition one commits to exclude intrinsic and short-lasting variations of alertness and extrinsic longer term variations of alertness, even though both seem perfectly possible. Second, for phasic alertness in fact, we can assume that it can be elicited not only by external stimuli but also by internal processes. For example, urgency (time-pressure) has been found to phasically boost stimulus-driven action, even in violation of current behavioral goals (Krause & Poth, 2023; Poth, 2021; Salinas et al., 2019). This observation resembles effects of alerting in cognitive control paradigms (Callejas et al., 2005; Fan et al., 2002; Fischer et al., 2010; MacLeod et al., 2010; McConnell & Shore, 2011; D. W. Schneider, 2018; 2019a; 2019b; Weinbach & Henik, 2012b) and in fact has been speculated to be due to phasic alertness (Poth, 2021). This is in line with neurophysiological findings showing that the LC-NE boosts supposed to underlie phasic alerting also occur in response to internal events, such as the outcomes of decision processes (Nieuwenhuis et al., 2005). Thus, in sum, we should assume an extrinsic form of phasic alertness that is elicited by external stimuli, but should also be open to intrinsic forms of phasic alertness triggered by internal events (estimated elapsed time in urgent tasks, Krause & Poth, 2023; Poth, 2021; Salinas et al., 2019).

#### Phasic alertness as a bottom-up modulation of perception and action

The term alerting has been used with considerable heterogeneity throughout the literature, sometimes referring to the process leading to phasic alertness (A. Petersen et al., 2017; Poth, 2020), but sometimes also referring to cuing attention to prioritize processing at a specific time (Coull et al., 2001), or to increasing the temporal expectation for stimulus occurrence (as discussed by Weinbach & Henik, 2012a). Therefore, it is often not clear which underlying mechanisms should be implicated for interpreting empirical results (Hackley, 2009; Weinbach & Henik, 2012a).

Phasic alertness is assumed to come from a bottom-up process driven by the stimulus of the alerting cue (for discussion, see Hackley, 2009; Poth, 2020). As such, it should depend on the physical characteristics of the stimulus, such as intensity, timing and duration, and its stimulus modality (e.g., Dietze & Poth, 2023). Congruent with this assumption, alerting effects on reaction times in choice tasks increase (up to a point) with increasing intensity of the alerting cue (Adams & Behar, 1966; Petersen et al., 2017; and increasing CTOAs, Dietze & Poth, 2023). Bottom-up phasic alertness should be relatively short-lived. It should be long-lasting enough to improve fast perception and action after a warning stimulus. But it should be short-lasting enough to regain susceptibility to subsequent warning stimuli and to prevent that alerting-induced impairments in resolving cognitive conflicts (or interference between cognitive processes) last long and lead to large detriments of behavior. Recent experiments show that alerting effects last at least for cue-target onset asynchronies of up to 1500 ms (Dietze & Poth, 2023). This may seem already quite long for striking the balance between supporting perception and action and impairing cognitive conflict resolution. For situations without cognitive conflicts, the duration of phasic alertness seems to be optimal for supporting the next action (Dietze, Recker, & Poth, 2023). Specifically, the next manual action in sequence of actions is speed up after an alert, whereas the next action after that falls already in the normal range of reaction times (Dietze et al., 2023).

In terms of stimulus modality, alerting traditionally has been assumed to be strongest for auditory stimuli (Bertelson & Tisseyr, 1969; Davis & Green, 1969; Harvey, 1980), in line with the idea that audition was the primary “warning sense” for humans (Dykstra et al., 2017). However, recent experiments reveal that audition and vision lead to similar alerting effects once their stimulus intensities are equated for processing speed based on the reaction times for a simple detection (Dietze & Poth, 2023). Thus, audition is not a primary warning sense with direct impact on phasic alertness. Rather, phasic alertness seems to be triggered qualitatively similar by the different sensory modalities. The differences in alerting effects seem to reflect low-level characteristics of the senses, such as their basic processing speed which is for example higher for audition than for vision (Recanzone, 2009; Torre et al., 1995).

#### Phasic alertness and top-down temporal preparation: Distinct processes?

Top-down temporal orienting of attention occurs when a cue indicates the time when the target stimulus for the current task was about to appear (Correa et al., 2010; Coull et al., 2001; Coull & Nobre, 1998). Similar to phasic alertness, temporal attention supports behavioral performance by reducing reaction times and accuracy in detection and discrimination tasks (Correa et al., 2004; 2005; 2010; Coull & Nobre, 1998). For temporal attention, participants must learn (by explicit instruction or implicit experience), that the target occurs at a specific time relative to the cue, so that processing at this time can be enhanced by attentional prioritization. What distinguishes temporal attention from phasic alertness is that temporal attention is flexible: A given cue could indicate that the target was going to appear shortly or after a longer interval (Correa et al., 2004; Coull & Nobre, 1998), and in both cases the cues can be used to support behavior. In contrast, phasic alertness is inflexible. It supports processing for a more or less ballistic time-window, so that processing after this time-window cannot be enhanced over the level within the time-window (Hackley, 2009; Hackley et al., 2009). After a manual action, the modulation of perception and action could be shut down, so that alerting effects are limited to only the next action (Dietze et al., 2023).

Temporal expectation supports behavior again akin to phasic alertness. It reduces reaction times in choice tasks (for a review, see Nobre et al., 2007), increases visual perceptual accuracy, and increases the speed at which visual information is encoded into visual short-term memory (Vangkilde et al., 2012; 2013). However, in contrast to phasic alertness, temporal expectation builds up over an experimental trial, according to the hazard rate at which targets occur after cues across the experiment (Vangkilde et al., 2012; 2013; for a discussion, see Weinbach & Henik, 2012a). The hazard rate specifies the probability that the target occurs now, given that it has not appeared up until now (Luce, 1991). For example, consider an experiment in which cue-target intervals range from 0.5 to 3 s, and in which all intervals within this range are uniformly distributed (i.e. equally probable). Across the experiment, participants thus learn that the more time has already elapsed during a trial, the higher was the probability that the target appeared now, eventually reaching certainty at an interval of 3 s. Probability distributions in which the hazard rate increases over time are said to be aging. In contrast, non-aging probability distributions of cue-target intervals have a constant hazard rate. This means that the probability that the target occurs at an instance, given it has not appeared before, is constant over trials (Luce, 1991; Näätänen, 1971; Näätänen et al., 1974). These distributions contain many more short intervals and fewer and fewer longer intervals. Examples for such distributions include the exponential and the geometric probability distribution, both of which have been used to control for the build-up of temporal expectation across trials (A. Petersen et al., 2017; Poth, 2020; Poth et al., 2014; Vangkilde et al., 2012; 2013; Weinbach & Henik, 2013). When cue-target intervals come from non-aging distributions, participants experience more often a short interval, and then less and less often a longer interval. Thus, they develop a strong expectation for short intervals, and with increasing interval duration, their expectation decreases. Now, evidence dissociating phasic alertness from temporal expectation comes from experiments using these non-aging probability distributions. Even though the cue did not predict the onset of the target stimulus, substantial alerting effects on choice reaction times (Lu et al., 2014; Poth, 2020; Weinbach & Henik, 2013), temporal perception thresholds, and the speed of visual processing (A. Petersen et al., 2017) have been found. These findings thus argue that temporal expectation and phasic alerting effects stem from different processing sources. However, at least one recent study found that if waiting times to a target in a condition without alert followed a non-aging probability distribution, choice reaction times still decreased with increasing waiting time (Dietze & Poth, 2023). These findings suggest that some form of temporal preparation still occurred, arguing that non-aging waiting times offer no guarantee that temporal expectation was controlled.

It is a crucial question whether one can dissociate phasic alertness from temporal expectation or temporal preparation more generally. It has been argued that the crucial difference between phasic alertness and temporal expectation is that the latter is based on temporal contingencies between cue and target stimulus (Weinbach & Henik, 2012a). However, we could speculate that it still reflected a rudimentary form of temporal preparation. The phasic alerting effect seems to develop most strongly at CTOAs below 500 ms (Fan et al., 2002; 2005; Hackley, 2009; Hackley et al., 2009) but seems to last at least up until 1500 ms (Dietze & Poth, 2023). These intervals could match the statistics of environmental cues that predict events relevant for action or action sequences (Dietze et al., 2023) in everyday life. Thus, through life-long learning, temporal preparation mechanisms could be established that then give rise to a phasic alertness that would be fast, occurred only within the learned time-window, and would otherwise seem bottom-up (note, however, that learning effects can be hard to categorize as top-down or bottom-up, Awh et al., 2012). This would argue that it is impossible to clearly dissociate phasic alertness from temporal preparation.

Another point against a distinction of phasic alertness and temporal preparation comes from the hierarchy assumed to be present in cognitive processing (e.g., Norman & Shallice, 1986; Posner, 1978). Specifically, it is assumed that cognitive processes happen in successive stages or phases, where one stage or phase received the processing result of the previous, which may be organized in processing episodes (Poth et al., 2015; Poth & Schneider, 2016; 2018; W. X. Schneider, 2013). Now, if the successive processing stages or phases operated at different processing speeds (e.g. due to temporal summation in neuronal processing, Zhou et al., 2018), alerts that have constant hazard for target appearance at the early stage could still deliver temporal information about target appearance at the later stage. For example, imagine that a non-aging distribution of cue-target intervals between 0 and 200 ms was encoded into the earlier stage, and from that stage, the later stage sampled information at 5 Hz (5 samples/s of 200 ms each). Then, all the possible waiting times for the target after the cue at the early stage would fall into one sampling cycle of the later stage. As a result, at the later stage, target appearance could be predicted and used to modify subsequent processing mechanisms. Thus, taken together, it may be impossible conceptually to dissociate phasic alertness from temporal preparation mechanisms completely (even in the absence of temporal contingencies, see e.g. Lawrence & Klein, 2013). We see that this calls for a change of strategy in phasic alertness research: Rather than asking *whether or not* it seems to offer more insight to ask *to what degree and how* phasic alerting effects were due to temporal preparation or expectation processes (experimental manipulations).

### How does phasic alertness depend on the current task?

If phasic alertness affected perception and cognition in a broad, non-specific manner and was driven by bottom-up stimulation and/or temporal expectation, we could assume that it was completely unrelated to the task set representing the current task with its stimuli and rules for action. The evidence regarding the relationship between phasic alertness and the current task set is mixed. Initially, it was found that the informativeness for directing spatial attention that was associated with the stimuli used as alerting cues affected how well the cues could elicit phasic alertness (Lin & Lu, 2016). This seems to suggest that the representation of the stimuli’s informativeness within the current task set influenced the stimuli’s alerting effects. Following up on this result, however, a recent study found that alerting was equally effective, irrespective of whether the stimuli used as alerting cues had been learned to be informative for guiding spatial attention (Dietze & Poth, 2022). Thus, it remains to be clarified whether or not the task’s stimuli represented in the current task set influence how phasic alertness can be induced once alerting cues belong to these stimuli.

Besides associations with a task’s stimuli, the pure occurrence of the stimuli used as alerting cues within the task set could influence how the cues can induce phasic alertness or, respectively, how alerting effects on reactions can become manifest. A recent study investigated the relationship between phasic alertness and surprise (Dietze, Horstmann, & Poth, 2024). After a baseline period of trials without alerting cues, the first alerting cue was shown as a surprise for the participants, after which it occurred on half of the trials (as in a standard alerting experiment). Initially, the surprising alerting cue impaired performance by prolonging participants’ reaction time. After a few trials, however, the classic alerting effect emerged as a benefit for participants’ reaction times. Surprising stimuli generally seem to hinder fast responses (Horstmann, 2015). Therefore, it seems that the effect of surprise overpowered or blocked the beneficial alerting effect from becoming manifest in reaction times. Surprise could lead to a general inhibition of responding (Meyer et al., 1991; Niepel et al., 1994), perhaps to facilitate orienting responses in new situations (see, e.g., Nieuwenhuis et al., 2011). Given that humans learn regularities fast, sometimes in a single trial, after the initial exposure the alerting cues should not have been surprising anymore (Horstmann, 2015). That the alerting effect emerged after the initial exposure suggests that once the alerting cues was expected (and thus represented in the task set), the hindering effect of surprise vanishes. Thus, the beneficial effects of phasic alertness seem to require the expectation of alerting cues. They require the representation of alerting cues in the current task set, because otherwise alerting effects would be rendered adversary to performance by the surprise.

Besides the absence of surprise due to the expectation of alerting cues, phasic alertness seems to require top-down expectation in another way. A recent study investigated whether alerting effects required that alerting cues predicted the occurrence of a target stimulus that participants had to respond to (Poth & Dietze, in prep.). If alerting cues predicted that a target was about to follow afterwards (with a probability of p = .9 across trials), the classic alerting effect was found, namely an improvement of reaction times by the alerting cues. However, if alerting cues predicted that no target was about to appear (i.e. targets only followed with a probability of p = .10), the cues had no effects on participants’ performance. Alerting cues predicting that targets appear shortly could be allowed access to modulating the state of phasic alertness for perception and action, leading to the beneficial effects on reaction times. This could be the case even if the alerting cues do not provide a precise temporal prediction of when the target occurred (cf. Grabenhorst et al., 2019; 2021). Rather, a qualitative association between alerting cues and target stimuli that predicts qualitatively that a target occurred could be sufficient for allowing alerting effects. In contrast, however, if alerting cues were associated with the absence of target stimuli, they should not be permitted to influence the current state of phasic alertness, which would explain the absence of alerting effects in this case.

The discussed findings suggest that even though phasic alertness may originate from bottom-up arousal, whether or not phasic alertness can be used to support actions within the current task requires top-down influences such as expectations about alerting cues and target stimuli. However, there also seem to be constraints of the beneficial effects of phasic alertness that are linked to overt actions more directly. A recent study investigated how alerting affected action in a complex task requiring sequential actions, namely a sequence of mouse clicks on numbered visual targets (Dietze et al., 2023). Auditory alerting cues were presented before the onset of the visual targets, and it was assessed how the alerting cues influenced the sequential actions as compared with a no-cue condition. Results revealed an alerting effect consisting in shorter reaction times on trials with alert compared with trials without alert. This alerting effect was limited to only the first action in the sequence, so alerting left all subsequent actions unaffected. Critically, this was the case irrespective of how much time was required for the individual actions, suggesting that the limitation of alerting effects to the first action was not due to the short-livedness of phasic alertness. Thus, these findings revealed that phasic alerting and action are related in a more fundamental way. For instance, the cognitive processes implicated in setting up a task set, response selection, and execution, etc., of the first action could allow for a speeding up of the action by bottom-up arousal from the alerting cues. However, the execution of the first action or the cognitive processing such as task-set reconfiguration, response selection etc. for the second action could shut off the arousal or could prevent it from reaching the cognitive mechanisms supporting task performance. In either way, this means that phasic alertness does not happen in a set time-window (Jankovic et al., 2022), but in episodes created by action.

In sum, the discussed findings reveal that phasic alerting does not work in a bottom-up fashion entirely. Instead, top-down expectations of the alerting cues and their association with target stimuli linked with the alerting cues are required to achieve alerting effects on performance. In addition, the beneficial effects of phasic alertness seem to unfold in action episodes, so that only the next action is supported, whereas subsequent actions are left unaffected.

## The quest for a mechanistic understanding of phasic alertness

We have discussed a number of problems that we must face to achieve a mechanistic understanding and computational models of phasic alertness. Now, we can sketch a research program for solving the outstanding problems that need to be addressed before we can develop computational models of the processes that underlie phasic alertness.

As explained above, it is a fundamental assumption that phasic alertness is a short-lasting state of the cognitive system that increases the readiness for perception and action by modulating diverse cognitive processes at multiple loci in the cognitive processing chain (as reviewed above, section *Phasic vs. tonic alertness: Two types of readiness for perception and action*). Even though this state-assumption is at the heart of our understanding of phasic alertness, it remains to be tested empirically. This is one of the most important challenges of our research program. We can address this challenge by making use of one of the problems outlined above. In the section *Extrinsic stimulus-driven versus intrinsic phasic alertness*, we have discussed how it is problematic that phasic alertness is studied while neglecting an individual’s level of tonic alertness, since alerting effects should depend on tonic alertness in an inverted-U-shaped function. Now, to address our challenge of establishing that alerting manipulations indeed change the current state of our cognitive system, we can make use of this relationship and arrive at a testable criterion identifying phasic alertness as a cognitive state: We manipulate or measure different tonic alertness levels in conjunction with our phasic alertness manipulation. For example, the tonic alertness level could be manipulated by asking participants to assume different body positions that have been found associated with specific states of tonic alertness (for instance, lying down, sitting, standing in a difficult-to-hold stance; Barra et al., 2015), while a typical phasic alertness experiment is performed. Likewise, tonic alertness could be manipulated by inducing relaxation states (e.g., by performing relaxation exercises such as progressive muscle relaxation, Rausch et al., 2006, that might be scaled in intensity by adapting the duration of the exercise), and/or higher-arousal states (e.g., by asking participants to perform a challenging task, before the phasic alertness experiment, or by including variable deadlines for responding that cannot be met on some catch trials, cf. Poth, 2021, that would be excluded from the data analysis). In these ways, the states of tonic alertness and the states of phasic alertness (e.g., a no alert condition, a low-stimulus-intensity alert condition, and a high-stimulus-intensity alert condition) could be crossed in factorial designs. Moreover, physiological measures of arousal (e.g. stimulus-independent pupil size, e.g. Poth, 2021; see, Mathôt, 2018, for a review) could be used to assess tonic alertness states during a phasic alertness experiment, so that one could correlate momentary tonic alertness states with phasic alerting effects. One could also assess participants’ objective state of tonic alertness by combining phasic alertness paradigms with tests of vigilance (as has been done by Luna et al., 2018). Last, one could also assess their subjective state of tonic alertness by asking about their mental states that fluctuate on the same time-scale as tonic alertness, such as perceived mental and physiological relaxation states and fatigue (Steghaus & Poth, 2022; Steghaus & Poth, 2024). Then, phasic alerting should only work at the intermediate levels of tonic alertness. In other words, if tonic alertness was extremely low (e.g. if one is fatigued or drowsy, Unsworth & Robison, 2016), phasic alerting manipulations should not suffice to elicit any alerting effects on behavior. Likewise, if tonic alertness was extremely high, any stimulation should be able to elicit a direct response, leading to many errors and premature responses (Esterman et al., 2014; Fortenbaugh et al., 2017). This pattern of alerting effects that were restricted to intermediate levels of tonic alertness identifies the alerting effects as a change of the current state of readiness for perception and action.

The second fundamental assumption about phasic alertness that remains to be tested empirically is that it operates prospectively and prepares for oncoming perception and action in an online fashion (Table 1). Alternatively, alerting cues could also be encoded with the to-be-processed signal or could travel the processing chain in parallel to this signal, and could then lead to the expression of alerting effects later on, when the signal is analyzed (see above). This would mean that alerting would not work online and prospectively, but would work in an offline fashion instead. One way to address the problem that the way of operation (the modulation of online vs. offline processing) is untested, is to incorporate several rather than a single alert in alerting experiments (Poth, 2020). This could provide us with a first, tentative criterion distinguishing online and offline alerting effects: Alerting cues that shortly follow one another should work in concert if they affected processing by preparing for perception and action in an online fashion (provided that the level of tonic alertness was right, so their combination would not lead to overarousal, see above). In contrast, if alerting worked in an offline fashion by modulating processing at a locus of analysis of the target signals, then having more than one alerting cue should strengthen processing of multiple times within the target signal and this should lead to interference between these time points, akin to the effects of dividing attention between multiple stimuli (e.g., Bundesen, 1990). In this case, alerting effects should be reduced, even though they may or may not be visible against experimental conditions without alerting cues (Poth, 2020).

**Table 1:** Testable criteria for assumed alerting mechanisms.


ASSUMPTION	CRITERION

Phasic alertness is a cognitive state of increased readiness for perception and action	Alerting effects should be confined to intermediate levels of tonic alertness, but disappear at low and high levels of tonic alertness

Phasic alertness prepares for perception and action in an online, not an offline fashion	Multiple alerting cues should work in concert, rather than interfering with another

Phasic alertness is both, short-lived and stimulus-driven and not driven intrinsically	Alerting by means of external stimuli should be independent of manipulations of the intrinsic short-lived state of readiness for peception and action. Otherwise, “extrinsic“ and “intrinsic“ phasic alertness should interact.

Phasic alertness is bottom-up, and not driven by temporal expectation	Alerting effects should also appear once we control for temporal expectation using non-aging cue-target intervals. However, alerting may still reflect a rudimentary form of temporal expectation, rendering this a weak criterion still containing ambiguity.

Phasic alertness is bottom-up, and independent from the current task and related stimulus expectations	Alerting effects should occur whether or not alerting cues were themselves expected or predicted that a target (rather than no target) was about to appear. Both of these assumptions have been falsified already (Dietze et al., 2024; Poth & Dietze, in prep.)

Phasic alertness affects processing at multiple loci in the chain of cognitive processing	Alerting effects on dependent variables representing a specific processing stage should combine (additively, linearly, or nonlinearly).


The third fundamental problem is that the current definition of phasic alertness conflates two hallmarks, namely that it is short-lived and that it is triggered by an external stimulus (see the section *Extrinsic stimulus-driven versus intrinsic phasic alertness* above; see also Table 1). Not all short-lived changes in the readiness for stimulus-driven action are triggered by external stimuli. Rather, it has been demonstrated that internal processes such as the build-up of urgency or time-pressure can lead to effects akin to the ones of alerting on performance in cognitive control tasks (Krause & Poth, 2023; Poth, 2021). It remains an open question whether internally and externally triggered changes of readiness for perception and action would tap into the same mechanisms of phasic alertness. This calls for experiments in which both, alerting by means of external stimuli and the short-lived intrinsic state of alertness are manipulated in parametric factorial designs. Similar to the ways in which tonic and phasic alertness could be manipulated in conjunction (see above), one could induce short-lived intrinsic states of higher alertness by asking participants to respond within a certain deadline (e.g., Poth, 2021), and of lower alertness by including long waiting times for a trial to start (akin to long waiting times for target stimuli in experiments on temporal expectation, Vangkilde et al., 2012; 2013).

Our next fundamental problem targets the conception of phasic alertness as a solely stimulus-elicited versus a task-driven or expectation-driven process (Table 1). While it is commonly assumed that alerting happens in a bottom-up stimulus-driven fashion (Hackley et al., 2009; 2009; Hackley & Valle-Inclán, 1998), it can be difficult to distinguish it experimentally from top-down temporal orienting of attention and temporal expectation (Weinbach & Henik, 2012a). As we have argued above (section *Phasic alertness and top-down temporal preparation: Distinct processes?*), alerting may always reflect at least a rudimentary form of temporal preparation, in line with a conception as a “temporal attention filter” implemented by the locus-coeruleus nor-epinephrine system in the brain (Aston-Jones & Cohen, 2005). Therefore, rather than asking whether or not alerting reflected mechanisms of temporal preparation, it seems much more fruitful to ask about the specific characteristics of these mechanisms. For instance, we could aim to specify to what degree alerting effects were dependent on the temporal contingencies between alerting cues and target stimuli that individuals learned across experimental trials (Weinbach & Henik, 2012a).

If phasic alertness was strictly stimulus-based, it should be completely independent of the current task set delivering the priorites for attention and of the stimuli and events expected within the current task. While there is preliminary evidence for the independence of phasic alertness from task-based attention priorities (Ásgeirsson & Nieuwenhuis, 2017; Dietze & Poth, 2022), this does not hold true for expectations regarding the stimuli that can occur within a task. If alerting cues are surprising (Dietze et al., 2024) or if they predict that no stimulus rather than a target stimulus is about to appear (Poth & Dietze, in prep.), then they do not elicit alerting effects. Therefore, phasic alertness is not solely based on the stimulus. The alerting state that arises as a result of the stimulus-elicited processing of the alerting cue could still be solely stimulus-driven, but whether or not the effects of alerting find expression in behavior presupposes that alerting cues are expected and part of the current task set, for instance, when they predict the target (Poth & Dietze, in prep.). An influential theory of the locus-coeruleus norepinephrine system that may implement the alerting mechanisms holds that there are two modes of processing that govern whether one focuses on the current task to exploit it for rewards versus whether one focuses less and less on the task, more susceptible for distraction, and explores the environment for new tasks (Aston-Jones & Cohen, 2005). Even if phasic alerting in itself was stimulus-driven, the expression of its effects could still depend in a qualitative fashion on the mode that the individual is in at the moment. Alerting cues that are expected and represented within the task set could be used to support task performance when one is in the exploitation mode. This would lead to our established alerting effects. In contrast, in the exploration mode, one is naturally disengaged from the task and searches the environment for new tasks and new sources of reward, so that any within-task effects of stimuli such as effects of alerting cues on task performance should be absent. In this mode, alerting cues could attract attention to themselves, and by that could attract attention to the task within which they are embedded. However, for the beneficial effects of alerting to emerge, the individual would have to first engage in the task, and then also experience at least one, possibly a few exposures to the alerting cues (Dietze et al., 2024) and the subsequent target stimuli (Poth & Dietze, in prep.). Therefore, even though phasic alertness as a state change may be stimulus-elicited and independent of the two modes of processing, the modes could still determine how phasic alertness becomes expressed in behavior.

Last, we have argued that phasic alertness exerts effects at multiple loci in the chain of cognitive processing (see section *Where is the locus of alerting effects in the cognitive processing chain?*). Now, to develop a mechanistic understanding of these effects, we suggest to use the same experimental paradigms to assess alerting effects on different dependent variables that have been established as proxies for cognitive processing at specific processing stages. For example, using a similar paradigm and the same stimuli and participants, alerting effects on measures of visual encoding in unspeeded tasks (Kusnir et al., 2011; A. Petersen et al., 2017; Wiegand et al., 2017) could be compared with those on reaction times in speeded tasks (Dietze & Poth, 2022; Fischer et al., 2010; Poth, 2020; D. W. Schneider, 2018). Reaction times reflect processes in addition to visual encoding, such as response selection (Fischer et al., 2010; Hackley & Valle-Inclán, 1999). Therefore, the comparison of alerting effects could shed light onto whether later stages just inherited alerting effects from earlier stages, whether alerting effects on earlier and later stages were additive, linearly combined, or nonlinearly combined. It should be noted, however, that different dependent variables, such as reaction times and accuracy, could be sensitive to different aspects of processing within the given processing stages (van Ede et al., 2012; but see Mulder & van Maanen, 2013). Such a situation would be more complicated, but could be addressed by combining the different dependent variables in a common mathematical model (e.g., a drift-diffusion-model, Mulder & van Maanen, 2013; for a general overview, see Ratcliff et al., 2016).

## Conclusions

We have seen that a number of conceptual and empirical problems need to be solved for us to be able to develop a mechanistic understanding of phasic alertness and consequently to arrive at models describing the phasic alerting process in computational terms. To summarize, we need to establish I) that phasic alertness is indeed a cognitive state that readies the cognitive system for perception and action in general, II) whether or not (or how) it operates in an online or offline fashion, III) if the short-lasting phasic alertness is exclusively based on external stimuli or whether it can also be internally triggered, IV), if phasic alertness was bottom-up and not influenced by top-down temporal expectation, V) if phasic alertness was bottom-up and not dependent on the current task and stimulus expectations. We discussed how these challenges can be addressed and developed concrete empirical criteria offering ends to do so. Applying the criteria will allow us to draw conclusions as to which cognitive processes are implicated in phasic alertness for perception and action. As such, this will pave the way for experiments elucidating how these processes unfold over time, which will then provide the basis for modeling the processes computationally.

## Data Accessibility Statement

There is no data, experimental computer code, or analysis computer code associated with this article, since the article is theoretical.
